# New ways of Communicating Science: Open Science and Open Access

**DOI:** 10.21470/1678-9741-2020-0336

**Published:** 2020

**Authors:** Eduardo Augusto Victor Rocha, Paulo Roberto B. Evora

**Affiliations:** 1 President of Brazilian Journal of Cardiovascular Surgery, São Paulo, Brazil.; 2 Department of Surgery and Anatomy Ribeirão Preto School of Medicine. Faculdade de Medicina de Ribeirão Preto da Universidade de São Paulo (FMRP-USP), Ribeirão Preto, SP, Brazil.

How do we tell our story? How to transmit this story for years and centuries? These are fundamental questions that allow the *Brazilian Journal of Cardiovascular Surgery* (BJCVS) to improve its content and communicate with society. Scientific communication consists of informing, educating, sharing and raising awareness about science topics. Science has changed a lot, and in recent years, important initiatives have sought to make society aware of the openness in science and what can be gained from all these changes. But after all, what is open science? And open access? And how has the BJCVS been working with all this? Well, first, what is meant about Open Science?

Open science represents a new approach to the scientific process, based on collaborative work and new ways of disseminating knowledge, using information technologies and new collaboration tools^[1]^. The main premise of open science is to make research results accessible to the public in digital format without restriction and to extend the principles of openness to the entire research cycle, promoting sharing and collaboration as soon as possible, implying a systemic change in the way science and research are done^[2]^.

Open science is a large umbrella that includes Open Access. And one of the major milestones on open access is the Budapest Open Access Initiative. Published in February 2002, the document is a statement of commitment to open access (peer-reviewed) that must be made available allowing anyone to read, copy, distribute, print, search or reference the full text of articles, collect them for indexing, enter them as data in software, or use them for any other legal purpose, without financial, legal or technical barriers that are not inseparable from the internet access itself.

The BJCVS, part of SciELO and so many other national and international databases, has been learning and changing its way of informing science, not only to its collaborators but also to the whole society. The key to progress is to be transparent and accessible and this is the main value that BJCVS has been working with great dedication in recent years, since being an open access journal consists of always learning and being at the forefront of scientific communication.

**Fig. 1:**
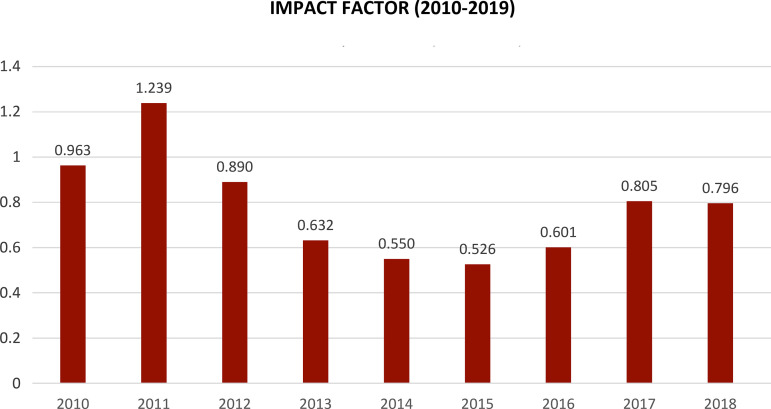
Impact factor (Journal Citation Reports) - 2010-2019.

Quality consists of a positive or negative degree of excellence and for BJCVS the improvement of positive qualities has yielded results such as the significant increase in citations received in recent years. In June 2020, the impact factor of the BJCVS was 1,053 (Figure 1).

However, there are some names worth mentioning. Dr. Domingo M. Braile made an enormous contribution to this victory. For 17 years (2003-2020), he was the editor-in-chief of BJCVS, being responsible for the organization and professionalization of our journal. The expansion in the number of articles and new communication channels increased the chances of more people reading our articles and citing them in their articles. We had 327 article submissions in the last year, of which 82.7% were foreign articles. In June 2020, we received 58 article submissions and the BJCVS was accessed more than 10,000 times.

Although BJCVS invests in new technologies to expand the dissemination of scientific content, its new scientific editor, professor Dr. Paulo Evora, with his extensive knowledge and scientific practice, has been transforming communication and its public engagement: through media communication channels (blog) and social media (Instagram). This stimulus to curiosity and creativity, in addition to the expansion of the network of potential collaborators, allows the dissemination of the results of our research to a larger number of people.

Open science, as well as open access, has impacted our status quo on how we do and communicate science. We have nothing to fear from this. As good researchers, we must always be open and accept changes that are welcome for a new world.
